# Atractylenolide I Induces Apoptosis and Suppresses Glycolysis by Blocking the JAK2/STAT3 Signaling Pathway in Colorectal Cancer Cells

**DOI:** 10.3389/fphar.2020.00273

**Published:** 2020-03-26

**Authors:** Yanxi Li, Yongpeng Wang, Zhexian Liu, Xingqi Guo, Ziwei Miao, Siping Ma

**Affiliations:** ^1^ Department of Colorectal Surgery, Cancer Hospital of China Medical University, Liaoning Cancer Hospital and Institute, Shenyang, China; ^2^ Department of Developmental Cell Biology, Key Laboratory of Cell Biology, Ministry of Public Health, and Key Laboratory of Medical Cell Biology, Ministry of Education, China Medical University, Shenyang, China

**Keywords:** atractylenolide I, colorectal cancer, apoptosis, glycolysis, JAK2, STAT3

## Abstract

Colorectal cancer (CRC) is the third most common cancer worldwide and is associated with a poor clinical outcome and survival. Therefore, the development of novel therapeutic agents for CRC is imperative. Atractylenolide I (AT-I) is a sesquiterpenoid lactone derivative of Rhizoma Atractylodis macrocephalae that exhibits diverse biological activities, including anti-cancer activities. However, the effects and potential mechanism of AT-I in CRC have yet to be fully elucidated. In this study, we aimed to examine the anti-cancer properties of AT-I and the associated functional mechanisms *in vitro* and *in vivo*. We found that AT-I treatment significantly suppressed the viability of CRC cell lines and inhibited colony formation, but to a lesser extent in NCM460 cells. Annexin V/PI staining showed that AT-I induced apoptosis in CRC cells, accompanied by increased caspase-3 and PARP-1 cleavage, enhanced expression of Bax, and reduced expression of Bcl-2. Furthermore, AT-I blocked cell glycolysis by inhibiting both glucose uptake and lactate production in CRC cells, and specifically downregulated the expression of the rate-limiting glycolytic enzyme HK2. In contrast, it had no discernable effects on the glycolytic enzymes PFK and PKM2. A mechanistic study revealed that AT-1 negatively regulates STAT3 phosphorylation through direct interaction with JAK2, thereby inhibiting its activation. Moreover, restoring the expression of STAT3 reversed the effect of AT-I on apoptosis and glycolysis in CRC cells. *In vivo* results revealed that AT-I significantly suppressed tumor growth in HCT116-xenografted mice. Collectively, our findings indicate that the anti-cancer activity of AT-I in CRC is associated with the induction of apoptosis and suppression of glycolysis in CRC cells, *via* the disruption of JAK2/STAT3 signaling. Our preliminary experimental data indicate that AT-I may have applications as a promising candidate for the treatment of CRC.

## Introduction

Colorectal cancer (CRC) is the third most commonly diagnosed cancer and the second leading cause of cancer-related deaths worldwide (Arnold et al., 2017; Bray et al., 2018). Recent statistics have indicated that 1.8 million CRC cases are diagnosed annually and that each year there are 881,000 CRC-related deaths worldwide (Bray et al., 2018). Despite the effectiveness of surgery, radiotherapy and chemotherapy, these strategies remain undesirable due to high rates of recurrence, drug resistance and potent side effects (Eng, 2009). Therefore, there is a pressing need to develop more effective preventive strategies and therapeutic drugs for CRC. Furthermore, a better understanding of the mechanisms underlying the anti-cancer effects of drugs is critical.

**Figure 1:**
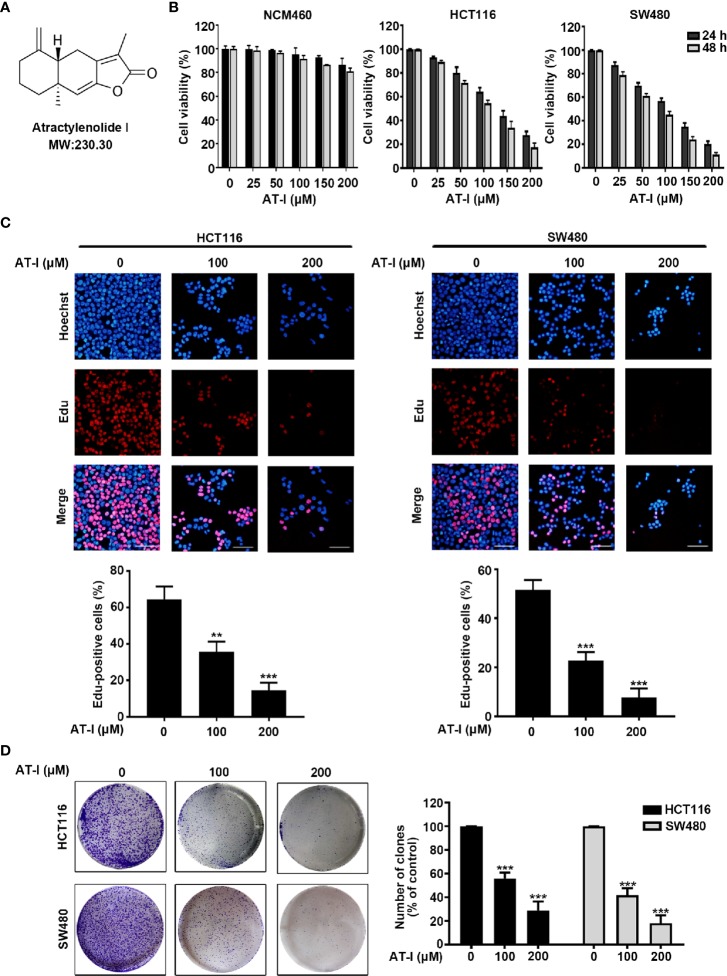
AT-I inhibits human CRC cell proliferation. **(A)** Chemical structure of AT-I. **(B)** Viability of NCM460, HCT116 and SW480 cells measured using the CCK-8 assay after treatment with different concentrations of AT-I for 24 or 48 h. **(C)** CRC cells were incubated with 0, 100, or 200 µM AT-I for 24 h, followed by further analysis using the EdU incorporation assay. Representative images are displayed. Scale bar = 100 μm. The EdU incorporation rate (the ratio of EdU-positive CRC cells to total Hoechst 33342-positive CRC cells) is shown. **(D)** Colony formation of CRC cells was determined following treatment with the indicated concentrations of AT-I. Left: representative images of the colonies. Right: statistical analysis showing the percentage of colonies relative to the control cells. **p < 0.01 and ***p < 0.001 versus the control group without any treatment.

Atractylenolide I (AT-I, chemical structure is shown in **Figure 1A**), is a eudesmane-type sesquiterpenoid lactone derivative of Rhizoma Atractylodis macrocephalae, which is a plant commonly used in traditional Chinese medicine (Zhu et al., 2018). Pharmacokinetic studies in rats have demonstrated that, after oral administration, AT-I is readily absorbed and persists for extended periods of time, suggesting good oral bioavailability (Wang et al., 2008). AT-I has a wide range of biological and pharmacological properties, including neuroprotective, anti-allergic, anti-inflammatory, and anti-cancer activities (Wang et al., 2009; Lim et al., 2012; More and Choi, 2017; Xiao et al., 2018). Previous studies have shown that AT-I is beneficial in treating various cancers through the regulation of different molecules and pathways. For example, AT-I has been shown to induce the G2/M phase of the cell cycle and cellular apoptosis in ovarian cancer cells by inhibiting the phosphatidylinositol 3-kinase/Akt/mammalian target of the rapamycin (PI3K/Akt/mTOR) pathway (Long et al., 2017). In addition, AT-I induces apoptosis in lung cancer cells, both *in vitro* and *in vivo*, *via* the mitochondrial-mediated apoptotic pathway (Liu et al., 2013). These findings indicate that AT-I has potential as a drug compound for cancer treatment. A previous clinical study has shown that oral administration of AT-I to gastric cancer cachexia for six weeks restores patient appetite performance status without any toxic effects (Liu et al., 2008). These studies indicate that AT-I is a safe and promising candidate for cancer treatment. Moreover, AT-I has been shown to reduce intestinal adenoma formation through elevating autophagic flux *via* a decrease in D-dopachrome tautomerase (Li et al., 2018). However, the effects of AT-I in CRC have yet to be clarified, and further investigations are required in order to determine the underlying mechanisms.

One of the hallmarks of all cancer cells is dysregulated energy metabolism (Cairns et al., 2011; Hanahan and Weinberg, 2011). Cancer cells preferentially utilize glucose *via* the glycolytic pathway rather than through the typical oxidative phosphorylation, which is known as the Warburg effect. This effect increases both glucose uptake and utilization to meet the high energy demands of cancer cells and also maintains cancer cell redox homeostasis, thereby contributing to the promotion of cancer cell growth (Bensinger and Christofk, 2012; Liberti and Locasale, 2016). Therefore, the disruption of this glycolytic pathway has become a major area of focus in the development of novel anti-cancer drugs, as exemplified by those strategies aimed at inhibiting key rate-limiting glycolytic regulatory enzymes, including hexokinase 2 (HK2), phosphofructokinase (PFK), or pyruvate kinase M2 (PKM2) (Scatena et al., 2008; Ganapathy-Kanniappan and Geschwind, 2013). Therefore, the inhibition of HK2, PFK, or PK to attenuate or suppress glycolysis in cancer cells is currently considered a potentially effective anti-cancer strategy (Pelicano et al., 2006). Identification of small-molecule inhibitors of these enzymes is a key priority in the development of compounds that could potentially promote a reduction in cancer cell proliferation, as well as an increase in cancer cell death.

In this study, we discovered that AT-I potentially inhibits CRC cell proliferation and induces CRC cell apoptosis. We also found that AT-I reduces HK2 expression and glycolysis in CRC cells, and that the mammalian target of the JAK2/STAT3 signaling pathway is crucial for the AT-I-mediated decrease in HK2 expression, glycolytic regulation, and cell apoptosis. Collectively, our results point to a novel mechanism whereby AT-I can exert therapeutic efficacy against cancer, potentially offering new opportunities for drug development.

## Materials and Methods

### Reagents and Antibodies

AT-I and AG490 were purchased from Selleck (Houston, TX, USA). Stock solutions of AT-I (100 mM) and AG490 (10 mM) were dissolved in dimethyl sulfoxide (DMSO). Antibodies against HK2, PKM2, PFK, JAK2, phospho-JAK2, STAT3, and phospho-STAT3 were purchased from Cell Signaling Technology (Beverly, MA, USA). Antibodies against caspase-3, PARP, cleaved caspase-3, cleaved PARP, Bcl-2, Bax, and β-actin were purchased from Abcam (Cambridge, UK).

### Cell Lines and Culture

The human CRC cell lines HCT116 and SW480 were purchased from the Shanghai Institute for Biological Sciences (Shanghai, China). Human normal colon mucosal epithelial cell line NCM460 was obtained from INCELL (San Antonio, TX, USA). These cells were cultured in Mycos’ 5A (HCT116), L15 (SW480) and DMEM (NCM460) media (Invitrogen, Carlsbad, CA, USA), respectively, supplemented with 10% FBS (Invitrogen, Carlsbad, CA, USA), 100 U/mL penicillin, and 100 μg/mL streptomycin in a humidified atmosphere of 95% air and 5% CO_2_ at 37°C.

### Cell Viability Assay

Cell viability was evaluated using a Cell Counting Kit-8 (CCK-8) assay kit (Dojindo Laboratories Tokyo, Japan). Briefly, the cells were seeded into 96-well tissue culture plates at a density of 1 × 10^4^ cells/well and treated with the indicated concentrations of AT-I. After 24 or 48 h of incubation, 10 μL of CCK-8 solution was added and then the cells were incubated for a further 1 h. The absorbance of cells at 450 nm was measured using a microplate reader (Bio-Rad Laboratories, Richmond, CA, USA).

### 5’-Ethynyl-2’-Deoxyuridine (EdU) Incorporation Assay

CRC cells were seeded in 96-well plates (5000 cells per well). After incubation for 24 h, the cells were pretreated with DMSO or AT-I for 24 h. Thereafter, 50 μM EdU (Ribobio, Guangzhou, China) was added to each well and following incubation at 37°C for 2 h, the cells were fixed with 4% formaldehyde for 30 min and then incubated with 2 mg/mL glycine for 5 min. After five washes with PBS, the cells were incubated with 100 μL of a 1× Apollo reaction cocktail for 30 min, following which, 1× Hoechst 33342 (5 μg/mL) was used to stain the nuclei for 30 min at room temperature. Cells were visualized in three fields of view/well under a fluorescence microscope (Carl Zeiss). The EdU incorporation rate was expressed as the ratio of EdU-positive cells to total Hoechst 33342-positive cells.

### Colony Formation Assay

CRC cells were treated with AT-I at the indicated concentrations and then transferred to a six-well cell culture plate at a density of 5 × 10^3^ cells/well. After further incubation for 14 days, the cells were fixed with 4% paraformaldehyde for 15 min and then stained with 0.1% (wt/vol) crystal violet. After incubation for 10 min, the cells were washed with PBS and then photographed using a digital camera. Colonies containing more than 50 cells were counted.

### 4, 6-Diamido-2-Phenylindole Hydrochloride (DAPI) Staining

Cells were stained with DAPI (Sigma-Aldrich, St Louis, MO, USA) to evaluate nuclear changes associated with apoptosis. Briefly, the CRC cells were seeded at a density of 1 × 10^5^ cells/well in six-well tissue culture plates, and then treated with the indicated concentrations of AT-I for 24 h. Thereafter, the cells were washed with PBS, followed by fixation with 4% paraformaldehyde for 30 min. After washing with PBS, the cells were stained with DAPI for 5 min. Samples were then mounted on a glass slide and the nuclear morphologies were observed using a BX51 fluorescence microscope (Olympus, Tokyo, Japan).

### Annexin V/Propidium Iodide (PI) Double Staining Assay

Apoptosis-mediated cell death was determined using an FITC Annexin V Apoptosis Detection Kit (BD Biosciences, San Jose, CA, USA) following the manufacturer’s guidelines. Briefly, CRC cells (1 × 10^5^ cells per well) were seeded in six-well tissue culture plates. After exposure to AT-I at the indicated concentrations for 24 h, both the attached and floating cells were collected. The cells were washed with PBS, then resuspended in 100 μL of binding buffer and stained with Annexin V-FITC and PI at room temperature for 15 min in the dark. Subsequently, 400 μL of binding buffer was added to each sample and the samples were examined using a FACScan flow cytometer (Becton Dickinson, Franklin Lakes, NJ, USA). Analysis of the data was performed using FlowJo software.

### Measurement of Glucose Uptake and Lactate Production

CRC cells (1 × 10^5^ cells/well) were seeded into six-well plates and treated with the indicated concentrations of AT-I for 24 h. The supernatant was collected and centrifuged to remove the cell debris. The glucose and lactate levels in the supernatant were measured using a glucose assay kit and a lactate assay colorimetric kit (Abcam, Cambridge, UK), respectively, according to the manufacturer’s instructions. Glucose consumption and lactate production were calculated based on a standard curve and normalized by cell numbers.

### STAT3 Overexpression Plasmid and Transfection

The human STAT3 expression plasmid pCMV3-STAT3-FLAG and empty vector pCMV3-C-FLAG were purchased from Sino Biological Inc. (Beijing, China). The CRC cells were transfected with the STAT3 overexpression vector or empty vector using Lipofectamine 2000 (Invitrogen, Grand Island, NY, USA) following the manufacturer’s instructions. After a 48 h transfection, the cells were treated with AT-I for 24 h and then harvested for analysis.

### Western Blot Analysis

Total cell lysates were obtained by lysing with RIPA buffer (Beyotime, Nanjing, China) containing protease inhibitors. The protein concentration was determined using the Bradford assay (Bio-Rad Laboratory, Inc., Hercules, CA, USA). Proteins from each sample were separated by electrophoresis and then transferred to a PVDF membrane. Following blocking for 1 h at room temperature, the membrane was incubated with a 1:1000 dilution of primary antibodies overnight at 4°C. The following day, the membrane was washed and incubated with HRP-conjugated secondary antibodies (1:5000; Abcam, Cambridge, UK). Protein bands were developed using ECL Prime Western Blotting Detection reagent (GE Healthcare, Buckinghamshire, UK), and images were obtained using an ECL chemiluminescence instrument (Tanon Science and Technology Co., Ltd., Shanghai, China). Quantification of protein band density was performed using Image J software. Signals were densitometrically quantified and normalized to β-actin expression.

### Molecular Docking

Autodock vina 1.1.2 was utilized for molecular docking to further study the interaction mode between AT-I and JAK2. The crystal structure of the JAK2 (PDB ID: 4BBE) was obtained from RCSB Protein Data Bank. The 3D structure of AT-I was constructed by ChemBio3D Ultra 14.0 software. The docking study was performed using the AutoDock Tools to generate the docking input files. Prior to docking, non-polar hydrogen atoms were merged and rotatable bonds were defined for the ligand. The search grid of the JAK2 site was identified as center_x: 3.581, center_y: -11.802, and center_z: -1.188 with dimensions size_x: 15, size_y: 15, and size_z: 15. AutoDock Tools and PyMol were used to visually analyze the docking results.

### Molecular Dynamics (MD) Simulation

The complex of JAK2 and AT-I constructed by the docking was used as the initial structure for the MD simulations. Amber18 package was used for MD. JAK2 and AT-I were edited with the ff14SB force field and the GAFF force field, respectively. Each simulation system was immersed in a truncated octahedral box of TIP3P explicit water, and the distance between the proteins and margin was extended 10 Å. To relax the structure, the system was energy-minimized during 5,000 minimization steps. After that, the system was gradually heated up to 300 K over 50 ps. This procedure was followed by 50 ps of NPT simulation at 300 K and 1 atm pressure to equilibrate the system. After equilibration, the systems were used to run 50ns long MD simulations. A time step of 2fs was used and coordinates of the system were saved every 20 ps. The resulting trajectory data were viewed and analyzed using the VMD software. Binding free energy was calculated by Molecular mechanics/generalized Born surface area (MM/GBSA).

### Tumor Xenograft Model

The animal experiments were approved by the Animal Ethics Committee of China Medical University (No. CMU2019321). For xenograft studies, male BALB/c nude mice (six weeks old and 18-20 g each) were injected subcutaneously with HCT116 (5 × 10^6^ cells/100 μl) into the right flank. When the diameter of the tumor reached to approximately 5 mm, mice were randomly divided into control and treated group (6 mice each group). Control group of mice was intraperitoneally injection with vehicle (0.9% sodium chloride plus 0.1% DMSO) and treated group was administrated with AT-I (50 mg/kg body weight) once daily for 3 weeks. The body weight and tumor volume were measured every 3 days after treatment begin. Tumor volumes were calculated using the formula: tumor volume (mm^3^) = (long diameter × short diameter ^2^)/2. At the end of the study, tumors were removed and then weighed.

### Immunohistological Analysis

For histological examination, dissected tumor tissues were fixed with 4% paraformaldehyde, embedded in paraffin and sectioned at 5 μm thicknesses. Levels of apoptosis in tumor tissue were determined using TdT-mediated dUTP-biotin nick end-labeling (TUNEL) assay with the in-situ Apoptosis Detection Kit (KeyGEN BioTECH, Nanjing, China) according to the manufacturer’s instructions. For immunohistochemical analysis of p-JAK2, p-STAT3 and HK2, the deparaffinized and hydrated sections were incubated with 0.3% H_2_O_2_ to block endogenous peroxidase activity and performed with sodium citrate buffer in a microwave for 20 min for antigen retrieval. Then the sections were incubated with the indicated antibodies overnight at 4°C followed by incubation with HRP-conjugated secondary antibody. 3,3′-Diaminobenzidine and hematoxylin were used to detect the immunocomplexes and nuclear counterstaining respectively. Finally, the tissue sections were captured with an Olympus digital camera attached to a light microscope.

### Statistical Analysis

All results were obtained from experiments that were repeated at least three times. For comparison of the two groups, statistical significance was analyzed with Student’s t-test. Statistical significance for multiple groups was performed using one-way ANOVA with Tukey’s *post hoc* test. Values were expressed as the mean ± standard error (SD). Statistical significance was set at P < 0.05.

## Results

### AT-I Suppressed Proliferation of CRC Cells

We initially investigated the potential anti-cancer properties of AT-I in CRC cell lines. We cultured two human CRC cell lines, HCT116 and SW480, in a medium containing 0-200 μM of AT-I for 24 and 48 h, and then evaluated cell viability using a CCK-8 assay. The results revealed that AT-I inhibited CRC cell viability in dose- and time-dependent manners, with half maximal inhibitory concentrations (IC50) of 126.8 μM and 97.19 μM for a 24 h treatment, and 98.49 μM and 70.44 μM for a 48 h treatment, respectively (**Figure 1B**). In addition, we also tested the cytotoxic effect of AT-I on normal cells NCM460. As shown in **Figure 1B**, AT-I exhibited as less cytotoxic against NCM460, with the IC50 values of more than 200 μM after 24 and 48 h treatment. To determine whether the decrease in viable cell density was due to an anti-proliferative effect of AT-I, we further measured the effect of AT-I on DNA replication using an EdU incorporation assay. The results showed that the numbers of EdU-positive cells were significantly reduced in both the SW480 and HCT116 lines after AT-I treatment for 24 h (**Figure 1C**). In addition, the anti-proliferative activity of AT-I was further evaluated using a colony formation assay. As shown in **Figure 1D**, there was a significant reduction in colony number after treatment with AT-I. Collectively, these findings indicate that AT-I inhibits CRC cell proliferation but has low cytotoxicity towards normal cells.

### AT-I Induced CRC Cell Apoptosis

**Figure 2:**
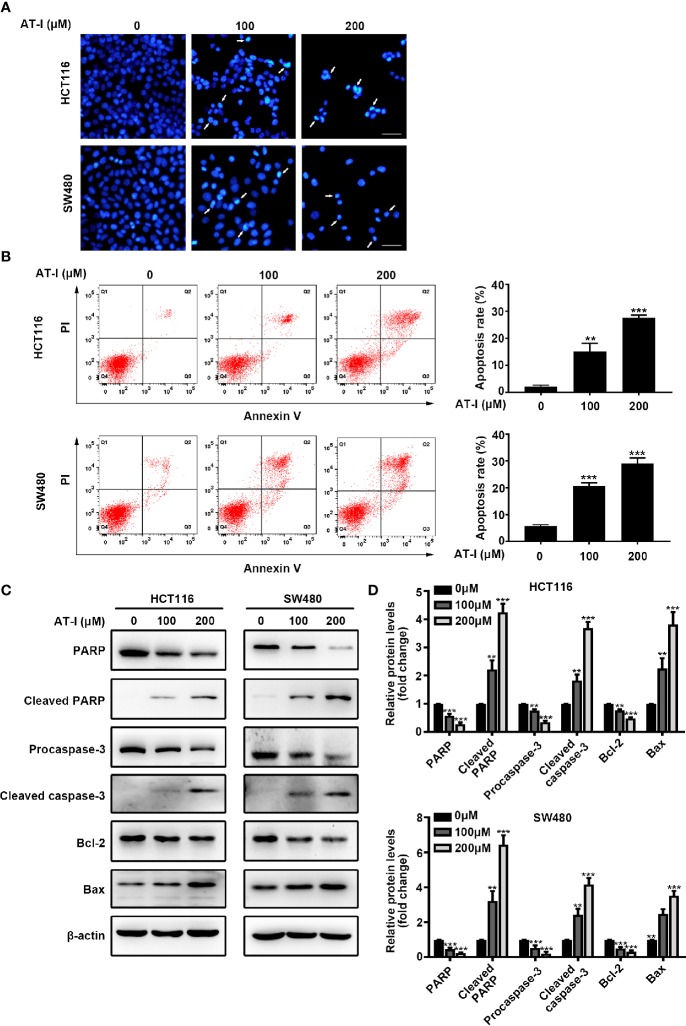
AT-I induces apoptosis of CRC cells. **(A)** DAPI staining of CRC cells treated using the indicated concentrations of AT-I for 24 h. White arrows indicate a nuclear morphological change in the treated cells. Scale bar = 100 μm. **(B)** Analysis of apoptosis using Annexin V-FITC/PI double-staining of CRC cells treated with the indicated concentrations of AT-I for 24 h. Apoptotic ratios represent the percentage of CRC cells in early apoptosis (PI negative/Annexin V-FITC positive) and late apoptosis (double-positive staining). Representative images of the results are shown in the left panel, and the results of the quantitative analysis are shown in the right panel. **(C)** Western blot analysis performed to detect the protein expression levels of PARP, cleaved PARP, procaspase-3, cleaved caspase-3, Bcl-2, and Bax in CRC cells after treatment with AT-I for 24 h. **(D)** The relative protein levels are normalized to those of β-actin, and these are shown as fold changes relative to the control group levels. **p < 0.01 and ***p < 0.001 compared with the control group without any treatment.

To determine whether AT-I-mediated inhibition of CRC cell proliferation is associated with apoptosis, we used DAPI staining to examine nuclear condensation (a characteristic of apoptosis). The results revealed prominent chromatin condensation and nuclear fragmentation in the AT-I-treated CRC cells (**Figure 2A**). To confirm the induction of apoptosis in response to AT-I treatment, we also assessed apoptosis using flow cytometric analysis after double labeling with Annexin V and PI. The results showed that AT-I treatment for 24 h increased the percentage of apoptotic CRC cells in a dose-dependent manner (**Figure 2B**). Given that activation of caspase-3 and cleavage of PARP are well-known molecular markers of apoptosis (Elmore, 2007), we next examined the levels of caspase-3 and PARP in CRC cells treated using AT-I. As shown in **Figures 2C, D**, AT-I triggered caspase-3 and PARP-1 cleavage as seen by the reduced expression of procaspase-3 and full length PARP and increased expression of cleavage of caspase-3 and PARP in a dose-dependent manner. An imbalance between the pro-apoptotic protein Bax and anti-apoptotic protein Bcl-2 would result in caspase-3 activation and subsequent apoptosis (Plati et al., 2011), and we found that AT-I dose-dependently downregulated Bcl-2 and upregulated Bax expression levels compared with the control group (**Figures 2C, D**). Collectively, these findings indicated that AT-I could efficiently induce apoptosis in CRC cells.

### AT-I Mediated Suppression of Glycolysis in CRC Cells *via* a Reduction in HK2 Expression

**Figure 3:**
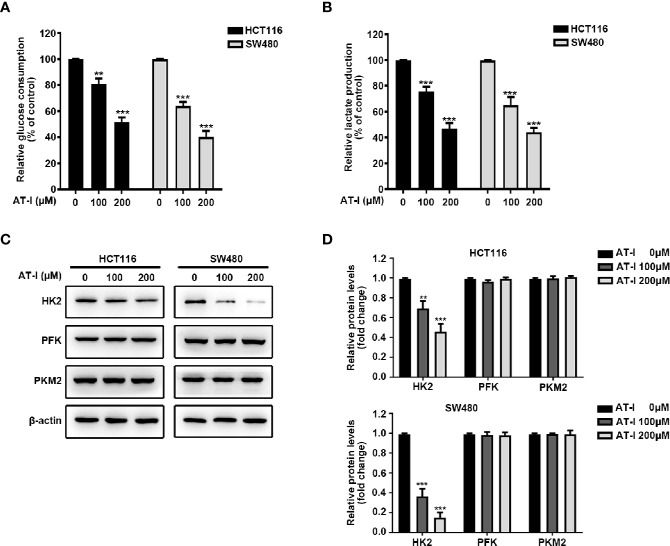
AT-I suppression of glycolysis in CRC cells *via* a reduction in HK2 expression. **(A)** Glucose consumption and **(B)** lactate production were measured in the CRC cells treated with the indicated concentrations of AT-I for 24 h. **(C)** Western blot analysis of key glycolytic enzymes [hexokinase 2 (HK2), phosphofructokinase (PFK), and pyruvate kinase M2 (PKM2)] and quantification of the results. **(D)** Protein levels were normalized to those of β-actin, and these are shown as fold changes relative to the control group levels. **p < 0.01 and ***p < 0.001 compared with the control group without any treatment.

Given that cancer cell proliferation and apoptosis are closely linked with glycolysis (Matsuura et al., 2016), we examined whether AT-I can be used to induce a metabolic shift in CRC cells. We found that after AT-I treatment, there was a significant dose-dependent decrease in glucose consumption by the CRC cells (**Figure 3A**). In addition, there was a significant decrease in lactate secretion in the AT-I-treated CRC cells (**Figure 3B**). Therefore, we examined the expression of three key rate-limiting glycolytic enzymes (HK2, PFK, and PKM2) in CRC cells treated with AT-I. Western blot analysis revealed that HK2 expression decreased in response to the AT-I treatment, whereas the expression of PFK and PKM2 remained unaltered (**Figures 3C, D**). Collectively, these findings indicate that AT-I may suppress glycolysis in the CRC cells by reducing HK2 expression.

### AT-I Inhibits JAK2/STAT3 Signaling Pathway in CRC Cells

**Figure 4:**
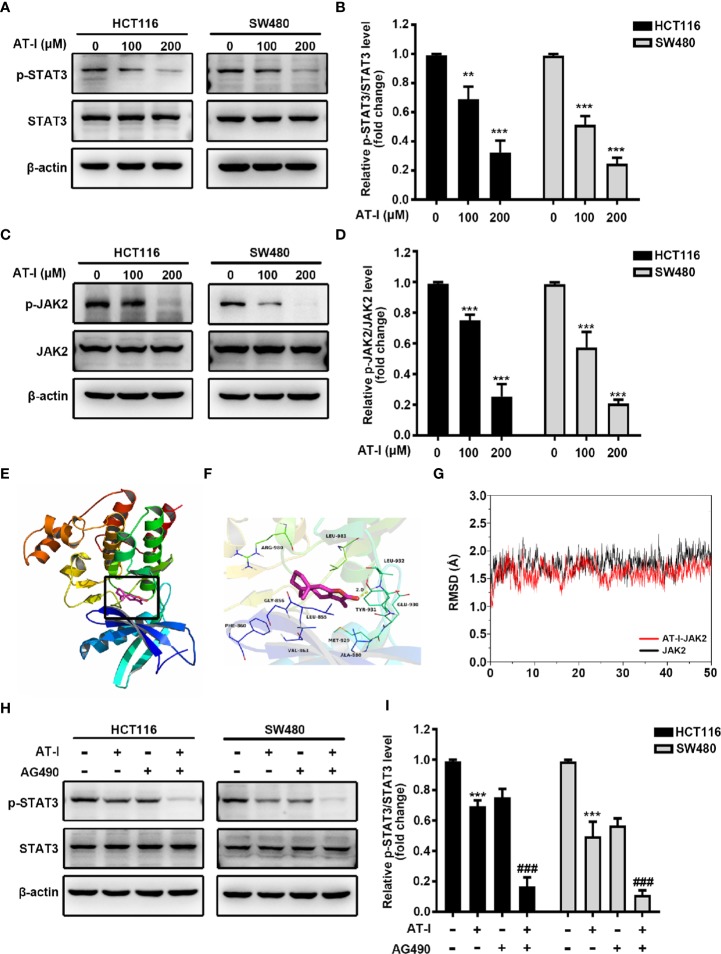
AT-I induced inactivation of JAK2/STAT3 signaling pathway in CRC cells. **(A)** Western blot analysis of p-STAT3 (Tyr705) and STAT3 in CRC cells treated with AT-I for 24 h. **(B)** P-STAT3 (Tyr705) levels were normalized to STAT3 levels, and these are shown as fold changes relative to the control group levels. **(C)** Western blot analysis of p-JAK2 and JAK2 and quantification of the results. **(D)** P-JAK2 levels were normalized to those of JAK2 levels and are shown as fold changes relative to the control group levels. **(E)** AT-I docking onto JAK2 using computer modeling. **(F)** Strong and stable hydrogen bonds between AT-I and Leu-932 of JAK2 (in yellow). **(G)** RMSD profile of AT-I complex with JAK2 and JAK2 Apo during the 50 ns MD simulation. **(H)** Western blot analysis of p-STAT3 and STAT3, after treatment with AT-I and AG490 as indicated.**(I)** P-STAT3 (Tyr705) levels were normalized to those of STAT3 and are shown as fold changes relative to the control group levels. **p < 0.01 and ***p < 0.001 compared with the control group without any treatment. ^###^*p* < 0.001 compared with the AT-I treated group.

Recent studies have shown that STAT3 is a transcription factor that regulates the transcription of HK2 (Jiang et al., 2012). Thus, we hypothesized that AT-I may regulate STAT3 activation in CRC cells. Phosphorylation of the Tyr705 residue of STAT3 appears to be an essential prerequisite for converting STAT3 to an active form (Levy and Lee, 2002). We found that AT-I suppresses the phosphorylation of STAT3 at Tyr705 in a dose-dependent manner without altering total STAT3 protein levels (**Figures 4A, B**). Considering the significant role of JAK2 in phosphorylation of the Tyr705 residue in STAT3 (Rawlings et al., 2004), we evaluated the activation of JAK2 in response to AT-I in CRC cells. As shown in **Figures 4C, D**, AT-I inhibited JAK2 phosphorylation in a dose-dependent manner. We therefore speculated as to whether JAK2 acts as a target of AT-I. To verify this hypothesis, we performed a computer docking analysis. The results showed that AT-I docks onto the binding site of human JAK2 (**Figure 4E**). AT-I was located at the hydrophobic site, surrounded by residues Leu-855, Phe-860, Val-863, Ala-880, Met-929, Leu-932, and Leu-983, forming strong hydrophobic binding. Importantly, we observed one key hydrogen bond interaction between AT-I and Leu-932 (bond length = 2.4 Å), which was the main interaction between AT-I and JAK2 (**Figure 4F**). All these interactions facilitated the anchoring of AT-I to the binding site of JAK2. Then we used MD simulations to evaluate the dynamic stability of the complexes of AT-I with JAK2. As shown in **Figure 4G**, the RMSD of docked AT-I-JAK2 complex and JAK2 apo all remain stable after a 25ns MD run. By contrast, the complex RMSD was more stable and lower than that of JAK2 apo. The total binding free energy between AT-I and JAK2 was -27.7kcal/mol, indicating a well binding between them. Collectively, these results indicated that AT-I undergoes a strong interaction with JAK2 and this binding interaction may increase the structural stability of JAK2. To confirm the role of JAK2 in AT-I-induced STAT3 inactivation, we pretreated CRC cells with AG490 (10 μM), a JAK2 inhibitor, followed by treatment with AT-I. We accordingly found that the AT-I-induced decrease in STAT3 phosphorylation was enhanced by pretreating the CRC cells with AG490 (**Figures 4H, I**). We therefore conclude that AT-I inhibits JAK2 activity and thereby functions as an inhibitor of the JAK2/STAT3 signaling pathway.

### Inactivated STAT3 Mediated the Inhibitory Activity of AT-I on the Glycolysis and HK2 Expression in CRC Cells

**Figure 5:**
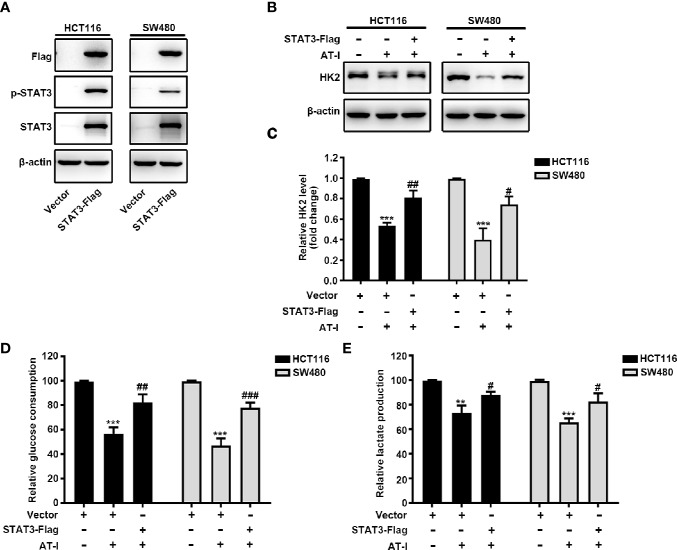
STAT3 inactivation is essential for AT-I-decreased HK2 expression and suppressed glycolysis in CRC cells. **(A)** The Flag-STAT3 plasmid was transfected into CRC cells, and after 48 h, these cells were treated with AT-I (200 μM) for a further 24 h, followed by western blot analysis to detect Flag, p-STAT3 (Tyr705) and STAT3 expression levels. **(B)** Western blot analysis of HK2 expression levels was performed for the CRC cells with STAT3 overexpression after AT-I treatment. **(C)** HK2 protein levels were normalized to those of β-actin and are shown as fold changes. **(D)** Glucose consumption and **(E)** lactate production were determined in CRC cells with the indicated treatments. **p < 0.01 and ***p < 0.001 compared with the control group without any treatment. *^#^p* < 0.05, *^##^p* < 0.01 and *^###^p* < 0.001 compared with the AT-I-treated group.

To further clarify the role of STAT3 in the regulatory effect of AT-I on glycolysis, we overexpressed STAT3 by transient transfection of a vector overexpressing STAT3 into CRC cells. After 24 h transfection, the expression of total STAT3 and STAT3 phosphorylation was increased remarkably in CRC cells (**Figure 5A**). Moreover, we found that when overexpression STAT3, AT-I-induced changes in the expression levels of HK2 were markedly reversed (**Figures 5B, C**). Further, we also observed that STAT3 overexpression rescued the deficient glucose consumption and lactate production in the AT-I-treated CRC cells (**Figures 5D, E**). These results indicated that AT-I-induced glycolysis suppression *via* decreased HK2 expression in CRC cells is partly mediated by inactivation of STAT3.

### Anti-proliferation and Pro-apoptotic Activities of AT-I Are Associated With STAT3 Inactivation in CRC Cells

**Figure 6:**
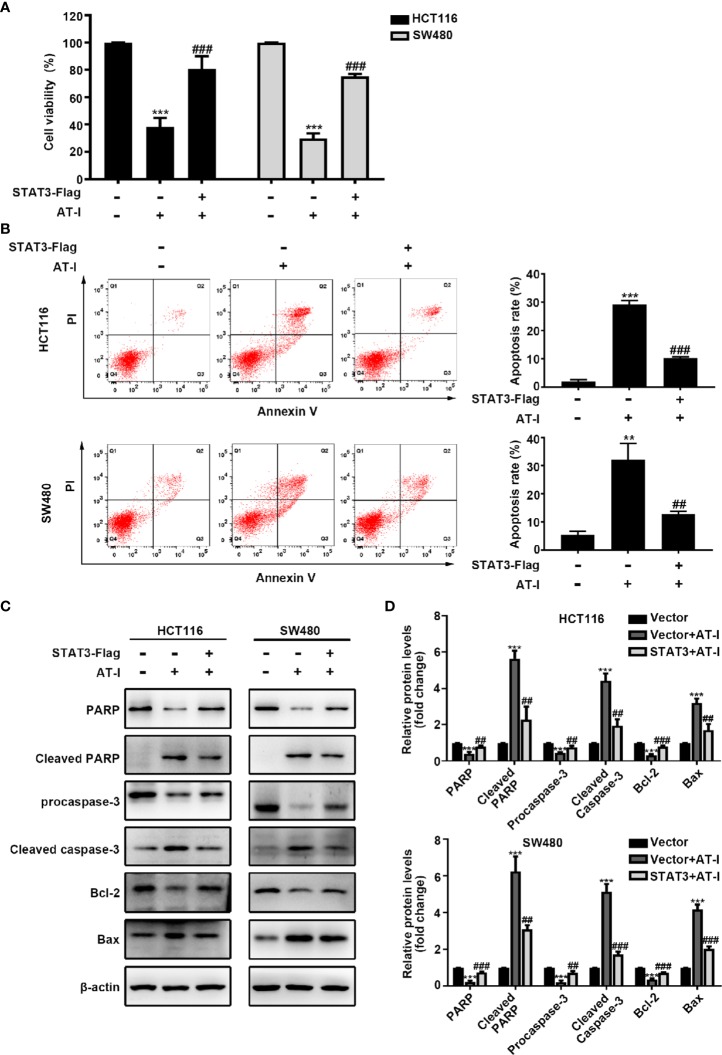
AT-I-induced STAT3 inactivation is involved in its pro-apoptotic activity in CRC cells. The Flag-STAT3 plasmid was transfected into CRC cells, and 48 h post-transfection, these cells were treated with AT-I (200 μM) for a further 24 h. **(A)** Cell viability determined using the CCK-8 assay. **(B)** Analysis of apoptosis by Annexin V-FITC/PI double staining. **(C)** Expression of apoptosis-related proteins detected by western blot analysis. **(D)** Protein levels were normalized to those of β-actin, and these are shown as fold changes relative to the control group levels. Data are shown as the mean ± SD of three independent experiments. **p < 0.01 and ***p < 0.001 compared with the control group without any treatment. *^##^p* < 0.01 and *^###^p* < 0.001 compared with the AT-I-treated group.

In addition to mediating glycolysis, STAT3 activation is also involved in the proliferation and anti-apoptosis of cancer cells [24]. We therefore wondered whether STAT3 is involved in AT-I-induced CRC cell apoptosis. After exogenously overexpressing STAT3 in CRC cells, the reduced cell viability caused by AT-I was partially reversed (**Figure 6A**). Moreover, the overexpression of STAT3 also reduced AT-I-induced apoptosis (**Figure 6B**). Consistent with these results, the effects of AT-I on apoptosis-related protein expression were significantly reversed after the overexpression of STAT3 in CRC cells (**Figures 6C, D**). These results indicated that STAT3 plays an important role in the anti-cancer effects mediated by AT-I in CRC cells.

### AT-I Inhibits Tumor Growth of CRC *In Vivo*

**Figure 7:**
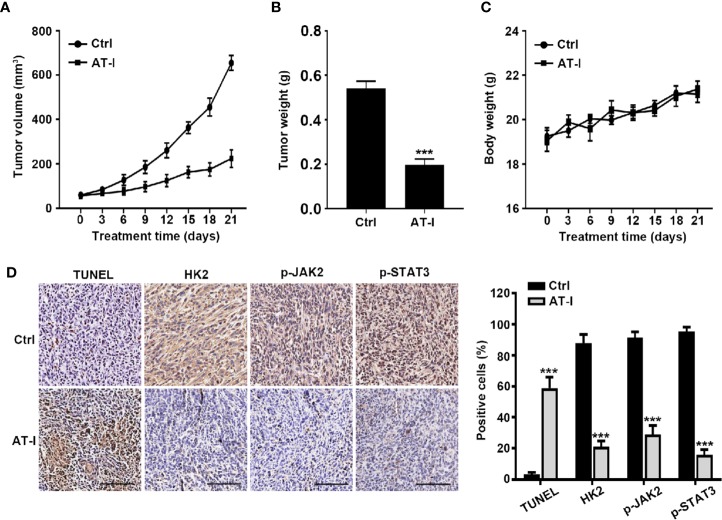
AT-I inhibits the growth of CRC cells in a nude mice xenograft Assay. Mice were administrated with vehicle or AT-I at 50 mg/kg/day for 3 weeks. Tumor volumes **(A)**, tumor weight **(B)** and body weight **(C)** of mice were measured. **(D)** Histological analysis of TUNEL, HK2, p-JAK2 and p-STAT3 levels in tumor tissue sections. Left: representative images of immunohistochemical analysis of tumor sections. Scale bar = 100 μm. Right: quantification of the percentage of positive cells in tumors. Data are represented as mean ± SD. ***p < 0.001 versus the Ctrl group.

In order to characterize the anti-cancer effects of AT-I *in vivo*, tumors of xenograft formed by the HCT116 cell were used. As shown in **Figure 7A**, during the three week treatment, AT-I significantly decreased tumor volume, when compared with the control group. Moreover, tumor weight of mice in AT-I treated group was markedly lower than that of the mice in the control group at the end of the treatment (**Figure 7B**). However, we observed that no significant difference in the body weight of mice between the control and AT-I-treated groups (**Figure 7C**), suggesting that AT-I has no obvious toxicity. Additionally, we detected apoptosis in xenografts mice using a TUNEL assay. We observed that AT-I increased the number of TUNEL-positive cells in the AT-I-treated group confirming that AT-I could induce apoptosis *in vivo* (**Figure 7D**). Consistent with the *in vitro* results, immunohistological analysis revealed that AT-I downregulated the expression of HK2, p-JAK2 and p-STAT3 in tumor tissuses of the xenograft mice (**Figure 7D**). Collectively, these *in vivo* data suggested that AT-I could induce CRC suppression through inhibiting JAK2/STAT3-dependent regulation of HK2 in tumors.

## Discussion

The current focus regarding the development of CRC treatment strategies is on discovering novel natural drugs with greater efficacy against cancer cells and lower toxicity toward normal cells (Rejhova et al., 2018). Rhizoma Atractylodis macrocephalae is a typical Chinese herbal medicine that has been reported to have diverse pharmacological effects (Zhu et al., 2018). A natural compound isolated from this plant, AT-I, has attracted increasing attention on account of its anti-cancer properties. In this study, we assessed the anti-cancer properties of AT-I against two CRC cell lines (HCT116 and SW480). We initially demonstrated that AT-I significantly inhibited the proliferation of tumor cells by using CCK-8, EdU and colony formation assay. However, AT-I showed comparatively less cytotoxicity towards the normal colon epithelial cell, indicating that AT-I selectively targeted CRC cells. This is encouraging for further research on AT-I as potential drugs against CRC.

Given that many types of cancer cell show disruption of the programmed death pathway, apoptosis represents a key therapeutic target in the treatment of cancer (Wong, 2011). Among the family of caspases, Caspase-3 has been regarded as a major executioner in apoptosis, and its activation promotes the down-streaming signal of PARP, subsequently contributing to cell death (Cohen, 1997). Usually the cleavage of PARP is used as an indicator of apoptosis (Wang et al., 1997). Here, we found that AT-I significantly induced cleavage of caspase-3. Subsequently, cleaved PARP was elevated. Mitochondria are also centrally involved in apoptosis (Wang, 2001). In response to diverse stimuli, Bcl-2 family plays a crucial role in initiating the intrinsic apoptotic pathway through modulation of anti-apoptotic (Bcl-2 and Bcl-xl) and pro-apoptotic (Bax and Bak) members (Wong and Puthalakath, 2008). Overexpression of bax is capable of promoting cytosolic accumulation of cytochrome c released from mitochondria and accordingly impels cell death. On the other hand, inhibition of cytochrome c release is governed by Bcl-2 (Ola et al., 2011). It has been proved that application of AT-I can altered the expressions of Bcl-2 family to promote cancer cells apoptosis (Liu et al., 2013; Long et al., 2017). Similar to previous studies, we found that AT-I markedly up-regulated Bax, whereas Bcl-2 was down-regulated in CRC cells. These results indicate that AT-I exhibits anti-CRC cancer activity by promoting apoptosis through regulating mitochondrial pathway.

As an important metabolic indicator, the Warburg effect facilitates cancer cell malignancy by increasing biosynthesis within cells, thereby promoting enhanced growth and proliferation (Tennant et al., 2010). In the present study, we assessed how AT-I alters glycolytic parameters in CRC cells and found that this agent inhibits both glucose uptake and lactate production. Overexpression of rate-limiting glycolytic enzymes, including HK, PFK, and PKM2, is a common characteristic of the Warburg effect (Mikawa et al., 2015). The HK2 isoenzyme is the form of HK that is most common in tumors, and is associated with tumor initiation and maintenance (Patra et al., 2013). PFK catalyzes the conversion of fructose-6-phosphate to fructose-1,6-bisphosphate and it is considered vital to cancer cell metabolic reprogramming (Al Hasawi et al., 2014). PKM2 regulates the first step of glycolysis, and is thus amongst the most important regulators of tumor cell metabolism (Christofk et al., 2008). Several natural compounds have been found to regulate glycolysis by targeting these glycolytic enzymes. For example, curcumin, a bioactive compound from the herb Curcuma aromatica Salisb, suppresses HK2 expression and leads to reduced glycolysis in CRC cells (Wang et al., 2015). Resveratrol, a polyphenolic compound, decreases cell viability and glucose metabolism by inhibiting PFK in breast cancer (Gomez et al., 2013). Shikonin, which is derived from Lithospermum erythrorhizon, has been shown to inhibit cancer cell glycolysis by specifically targeting PKM2 (Chen et al., 2011). Given the importance of these enzymes, we assessed their expression in CRC cells upon AT-I treatment and found that downregulation of HK2 expression in response to treatment with AT-1 had no discernable effect on PFK or PKM2. These results indicated that AT-I can suppress CRC cell glycolysis by reducing the levels of HK2 expression.

Previous studies have provided evidence that indicates an association between aberrant phosphorylation of the transcription factor STAT3 and a wide variety of cancers (Yu et al., 2014). STAT3 regulates the expression of essential genes that mediate cancer progression (Bromberg and Darnell, 2000; Haura et al., 2005). Recent studies have reported that STAT3 might play a crucial role in maintaining glycolysis in cancer cells by regulating HK2 (Li et al., 2015; Liu and Yu, 2018). In CRC cells, using STAT3 phosphorylation inhibitor cryptotanshinone could significantly decrease the HK2 expression (Du et al., 2019). Also, siRNA-knockdown STAT3 in CRC cells decreased mRNA levels of HK2 by reducing STAT3 recruitment to HK2 promoter (Du et al., 2019), consistent with the findings in breast cancer cells (Jiang et al., 2012). On the basis of these observations, we assessed the importance of STAT3 signaling with regards to the ability of AT-I to mediate anti-cancer effects in CRC cells. Consistent with previous findings, we found that STAT3 phosphorylation in CRC cells was markedly reduced in response to AT-I treatment, but did not alter the expression of total STAT3. JAK2 is a well-known upstream regulator of STAT3 activation in cancer cells (Bowman et al., 2000), and the JAK2/STAT3 pathway has been shown to alter the expression of key oncogenes and tumor suppressor genes to enhance the growth, survival, and metastasis of CRC cells (Xiong et al., 2008). Previous studies have reported that AT-I can inhibit JAK2 activation, indicating that the AT-I-induced downregulation of STAT3 phosphorylation is associated with changes in JAK2 (Fu et al., 2018). Consistent with these findings, we also found that AT-I can markedly reduce JAK2 phosphorylation in a dose-dependent manner. Moreover, several studies have reported that JAK2 can directly or indirectly interact with several phytochemicals and thereby mediate chemoprotective and anti-carcinogenic properties. We hypothesized that AT-I could directly interact with JAK2 to mediate its anti-cancer activity. On the basis of our computational docking study and dynamic stimulations, we predict that AT-I binds with the kinase domain of JAK2 *via* stable hydrogen bonding, which may be partially responsible for the anti-JAK2 activity of AT-I. Both AT-I and AG490 can reduce the levels of p-STAT3 in CRC cells and, as expected, the combination of these two agents appears to have more pronounced effects on p-STAT3 than their single administration. Moreover, STAT3 overexpression significantly attenuated the AT-I-induced inhibition of glycolysis and apoptosis. For *in vivo* experiments, we used HCT116 xenograft model, which has a high sensitivity to drug stimulation (He et al., 2016; Yang et al., 2016; Zhang et al., 2017). As reported previously, AT-I presented anti-cancer activity at a concentration of 25 to 75 mg/kg (Yu et al., 2016). Here, AT-I was used at 50 mg/kg to treat mice models. It showed satisfactory antitumor activity, as proved by tumor growth and immunohistochemistry assay. *In vivo*, AT-I significantly inhibited tumour growth and reduced the expression of HK2, phosphorylated JAK2 and STAT3. These findings indicate that AT-I may inhibit the JAK2/STAT3 signaling pathway by directly interacting with JAK2 and exerting its activity against CRC cells by inducing apoptosis and metabolic shifts.

In summary, the results of this study showed that AT-I suppresses CRC cell growth *in vitro* and *in vivo*. The anti-cancer properties of AT-I can be attributed to apoptosis and suppression of glycolysis, which, in turn, are mediated *via* AT-I-induced downregulation of HK2. Finally, the anti-cancer efficacy of AT-I in CRC is essentially mediated by inactivation of the JAK2/STAT3 signaling pathway. The findings of this study provide insights that can provide a basis for the potential development of a JAK2/STAT3 signaling inhibitor, which could be administered in combination with other drugs for the therapeutic treatment of CRC.

## Data Availability Statement

The raw data supporting the conclusions of this article will be made available by the authors, without undue reservation, to any qualified researcher.

## Ethics Statement

All protocols involving animal experiments were in accordance with the Care and Use of Laboratory Animals and approved by Animal Ethics Committee of China Medical University.

## Author Contributions

ZM and SM, the corresponding authors, conceived and designed the research. YL, YW, ZL, and XG performed the experiments, analyzed data, and prepared figures. YL, YW, and ZM drafted this manuscript. All authors contributed to manuscript and reviewed and approved the manuscript.

## Funding

This work was funded by Clinical Capability Construction Project for Liaoning Provincial Hospitals (LNCCC-D44-2015), and Natural Science Foundation of Liaoning Province (201602447).

## Conflict of Interest

The authors declare that the research was conducted in the absence of any commercial or financial relationships that could be construed as a potential conflict of interest.
